# Concentration-dependent oligomerization of an alpha-helical antifreeze polypeptide makes it hyperactive

**DOI:** 10.1038/srep42501

**Published:** 2017-02-13

**Authors:** Sheikh Mahatabuddin, Yuichi Hanada, Yoshiyuki Nishimiya, Ai Miura, Hidemasa Kondo, Peter L. Davies, Sakae Tsuda

**Affiliations:** 1 Graduate School of Life Science, Hokkaido University, Sapporo 060-0810, Japan; 2 Bioproduction Research Institute, ioproduction Research Institute and OPERANDO Open Innovation Laboratory, National Institute of Advanced Industrial Science and Technology (AIST), Sapporo 062-8517, Japan; 3 Protein Function Discovery Group and Department of Biomedical and Molecular Sciences, Queen’s University, Kingston, ON, K7L 3N6, Canada

## Abstract

A supersoluble 40-residue type I antifreeze protein (AFP) was discovered in a righteye flounder, the barfin plaice (bp). Unlike all other AFPs characterized to date, bpAFP transitions from moderately-active to hyperactive with increasing concentration. At sub-mM concentrations, bpAFP bound to pyramidal planes of ice to shape it into a bi-pyramidal hexagonal trapezohedron, similarly to the other moderately-active AFPs. At mM concentrations, bpAFP uniquely underwent further binding to the whole ice crystal surface including the basal planes. The latter caused a bursting ice crystal growth normal to *c*-axis, 3 °C of high thermal hysteresis, and alteration of an ice crystal into a smaller lemon-shaped morphology, all of which are well-known properties of hyperactive AFPs. Analytical ultracentrifugation showed this activity transition is associated with oligomerization to form tetramer, which might be the forerunner of a naturally occurring four-helix-bundle AFP in other flounders.

Organisms inhabiting ice-laden environments produce various cryoprotective agents to safeguard against freezing damage. Among them, antifreeze proteins (AFP) function by binding seed ice crystals to inhibit their growth^1^. The AFPs from fish, insects, plants, and microorganisms are structurally diverse macromolecules that have recently been grouped into either moderately active or hyperactive types. The crucial distinction for the latter is that they can bind the basal plane of ice^2^. This property accounts for their ∼10 fold higher antifreeze activity^3^, and is a key assessment made about newly discovered AFPs^4^,^5^,^6^,^7^.

A seed ice crystal, the target ligand of AFP, is composed of ordered water molecules in a hexagonal lattice, which is defined by three equivalent *a*-axes (*a*_*1*_, *a*_*2*_ and *a*_*3*_) perpendicular to the *c*-axis^8^. The orientation and vector of an ice plane in the hexagon are specified by Miller-Bravais indices^9^. For example, (0001) represents the two equivalent basal planes normal to the *c*-axis, (10–10) are the six equivalent primary prism planes, and (20–21) are pyramidal planes bound by one AFP type that produces a 12-sided hexagonal bipyramid with a 3.3:1 ratio of length to width^10^.

Regardless of the AFP type, their binding to ice causes microcurvature of the ice front between the surface-bound-AFPs through the Gibbs-Thomson effect^11^,^12^. This phenomenon depresses the non-equilibrium freezing temperature (*T*_f_) and slightly elevates the melting temperature (*T*_m_)^13^. The resultant difference between *T*_f_ and *T*_m_ is defined as thermal hysteresis (TH), which reflects the strength of ice-growth inhibition by an AFP. Moderately active AFPs require mM concentrations to achieve a TH activity of approximately 1 °C. Hyperactive AFPs reach this TH value at one tenth of these concentrations^3^. Another distinguishing observation is that ice crystals formed in moderately active AFP grow into their bipyramidal shape as growth-terminated ice planes become facets. Whereas, hyperactive AFPs shape ice during the melting process after initial snap-freezing. This difference in ice binding ability influences the way ice crystals ‘burst’ at *T*_f._ With moderate AFPs rapid ice growth occurs from the two tips of the bipyramid (i.e. basal planes) along the *c*-axis. In contrast, hyperactive AFPs direct explosive ice growth at *T*_f_ perpendicular to the *c*-axis^2^. The ice planes to which AFPs bind can be determined on a single ice hemisphere^12^ facilitated by the fluorescence-based ice plane affinity (FIPA) method^14^.

Type I AFP was initially discovered as a mixture of small (3–4 kDa) alanine-rich isoforms in winter flounder (*Pseudopleuronectes americanus*) plasma^15^,^16^. Among them, the 6^th^ isoform to elute from reversed-phase HPLC (HPLC-6) was the first AFP to have its crystal structure solved, which is a single amphipathic α-helix^17^,^18^. The three 11-residue repeats of type I AFP HPLC-6 bind ice through a hydrophobic surface comprising regularly spaced Thr and Ala residues repeating 16.5 Å apart on one side of the α-helix^19^. This ice-binding site binds the (20–21) pyramidal plane where the oxygen atom spacing is 16.7 Å^20^ and most likely does this through the organization of a clathrate water network around the hydrophobic methyl groups that is anchored to the nearby Thr hydroxyls and backbone peptide groups^21^,^22^. A second set of type I AFP isoforms were found in the skin and other peripheral tissues of this flatfish^16^. They are also single amphipathic α-helices. Later, a third isoform was discovered in winter flounder plasma, which is a 33-kDa homodimer with hyperactivity (type Ih)^6^. X-ray crystallography showed that this large isoform (Maxi) folds as a four-helix bundle with the putative ‘ice-binding’ residues projecting inwards to help organize ∼400 waters in its core that form a polypentagonal network to secure the four helices together^23^. The organized waters extent from the core to the surface of the protein where they are able to bind Maxi to multiple planes of a single ice crystal hemisphere.

The barfin plaice, *Liposetta pinnifasciata*, is one of the righteye flounders living in the Okhotsk coastal area of Japan. At maturity it is 30–50 cm in length and has a straight lateral line with black spots on the caudal fin (Fig. 1a). This fish synthesizes type I AFP as a mixture of isoforms that have been assessed for their cell-preservation ability^24^. Here we identified a 40-residue major isoform from this mixture (bpAFP), and examined its structure, function and biochemical properties. The bpAFP initially seemed to be an ordinary type I AFP, except for its extraordinary high solubility. However, it was noted that bpAFP was significantly more active and had a concentration-dependent ability to bind all over a single ice crystal hemisphere. Evidence presented here suggests that bpAFP can oligomerize in solution to resemble Maxi in having a four-helix bundle structure that confers hyperactivity.

## Results

### Muscle homogenate is a rich source of bpAFP

**Figure 1:**
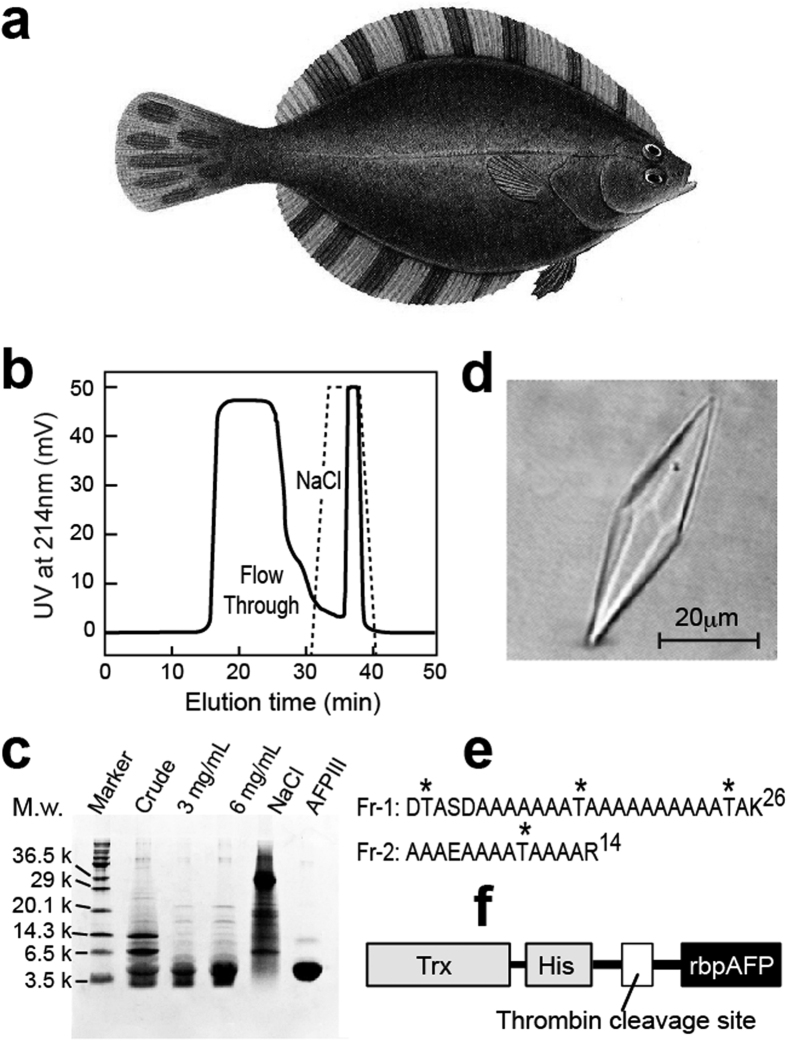
Purification of AFP from barfin plaice and bpAFP sequence: (**a**) Illustration of *Liposetta pinnifasciata* (barfin plaice) copied with permission from ‘An Illustrated Book of Fish’ (p.922, #3688), Hokuryukan Co. Ltd, Tokyo, Japan. (**b**) DEAE anion-exchange chromatography of partially purified bpAFP, where the AFP appeared in the large flow-through peak and contaminants were separately eluted with NaCl (dashed line). (**c**) Electrophoretogram of fractions from the anion-exchange chromatography step separated by 15% Tris-Glycine SDS-PAGE. Samples analyzed were the starting material for the chromatography (crude); two loadings of the flow through peak; contaminants eluted by NaCl; and type III AFP used as a molecular weight standard for 7 kDa. (**d**) Hexagonal trapezohedral ice crystal formed in 0.25 mg·mL^−1^ of the bpAFP solution. (**e**) Sequence of the two tryptic fragments (Fr-1 and -2) obtained from the HPLC-purified bpAFP, where asterisks mark threonines with the 11-residue periodicity. (**f**) Schematic diagram of the fusion protein construct composed of thioredoxin (Trx), His-tag, and a thrombin cleavage site used for the expression of rbpAFP.

Type I AFPs are typically isolated from the plasma of winter-caught righteye flounders. Here we have demonstrated the usefulness of fish muscle homogenate as the starting material for mass-purification of the native AFP that avoids the need to handle and bleed live fish. Approximately 6 g of low molecular weight (<30 kDa) protein was extracted from 2 kg muscle paste, and from this extract approximately 1 g of native bpAFP of 95% purity was recovered (Fig. 1b and c). The barfin plaice AFP changed the morphology of a single ice crystal into hexagonal trapezohedron (Fig. 1d) at a concentration of 0.25 mg·mL^−1^, which is distinct from the hexagonal bipyramid observed for wfAFP^17^.

### BpAFP is a type I AFP showing ultra-high solubility

The barfin plaice AFP was separated into several fractions by reversed-phase HPLC (Supplementary Figure S1). The one that showed the highest UV absorbance at 214 nm was digested into two fragments (Fr-1 and Fr-2) with trypsin (Fig. 1e) and sequenced. The N-terminal fragment is 26 residues long, and the C-terminal fragment, with an amidated C-terminus, is 14 residues long. D^1^TASDAAAAAAATAAAAAAAAAATAKAAAEAAAATAAAAR^40^-NH_2_ is the full primary sequence of this isoform. This is a typical type I AFP sequence composed of 3 tandem repeats of the 11-residue consensus sequence TX_10_ (where X is mostly alanine). However, bpAFP is more alanine-rich (75%) than other type I AFP isoforms that are typically only two thirds alanine. The extra alanines, up to 10 in a row, take the place of Asn and Leu that are not thought to play a critical role in either structure or function of type I AFP. The N-terminal D^1^TASD sequence and C-terminal amidation that form the α-helix capping structures are well conserved^17^,^25^.

**Figure 2:**
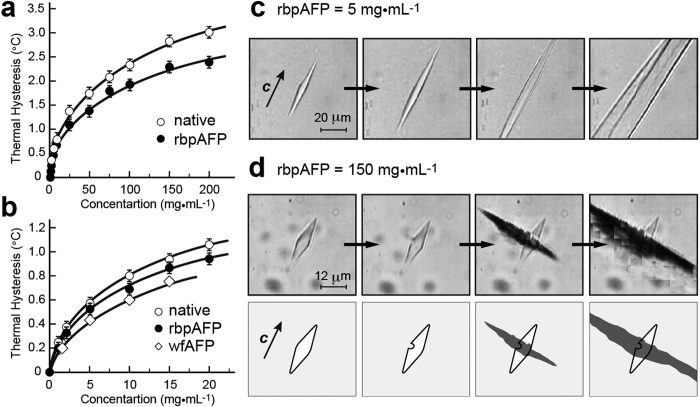
Ice binding ability and burst pattern of bpAFP: TH plot as a function of the concentration of native bpAFP (open circles) and rbpAFP (black dots) in the range of (**a**) 0–200 mg·mL^−1^ and (**b**) 0–20 mg·mL^−1^. The TH values of ordinary type I AFP (wfAFP)^35^ is plotted in panel (**b**) for comparison (open squares). (**c**) Bursting ice crystal growth along the c-axis observed at the TH limit of 5 mg·mL^−1^ of rbpAFP. (**d**) Bursting ice crystal growth perpendicular to c-axis observed for 150 mg·mL^−1^ of rbpAFP with illustrated interpretations.

A recombinant version of bpAFP (rbpAFP) was prepared from a fusion protein tagged with thioredoxin^26^ (Trx) (Fig. 1f), and used to ensure homogeneity of sequence. RbpAFP contains a C-terminal glycine following Arg40, which is the residue used in the native protein for amidation of the C-terminal Arg. It also has four-residue GSAM extension at the N teminus left after removal of the Trx fusion by thrombin. The concentration dependence of TH for both rbpAFP and native sample showed the typical hyperbolic profile seen with most AFPs (Fig. 2a). The TH values of the native bpAFP sample are slightly higher than those of the rbpAFP. Isoform mixtures of native samples are generally more potent than individual isoforms^27^,^28^. Both rbpAFP and native sample showed ultra-high solubility of approximately 650 mg·mL^−1^, which was determined spectrophotometrically (Nanodrop-1000, Thermo Scientific, USA) for the supernatants of their saturated solutions.

### BpAFP shows the evidence of hyperactivity

RbpAFP and bpAFP exhibited ∼1 °C of TH activity at the concentration of 10 mg·mL^−1^, while the TH increased to 2.5–3 °C with increasing the concentration up to 200 mg·mL^−1^ (Fig. 2a and b). Such a high TH value has only been recorded with hyperactive AFPs^2^,^3^. In contrast, with winter flounder type I AFP, <1 °C of TH was obtained at 20 mg·mL^−1^, which is close to its solubility limit^6^. Extrapolation of the hyperbolic profile of TH vs winter flounder AFP concentration would put an upper limit of ∼1.5 °C to its TH if this was not limited by protein solubility.

A TH activity of >2 °C is only one indication of hyperactivity. Another is the ‘bursting pattern’ of an ice crystal observed when TH is exceeded. At low rbpAFP concentrations (5 mg·mL^−1^) bursting occurs along the *c*-axis of the ice trapezohedron to give needle-shaped crystals (Fig. 2c). At high rbpAFP concentrations (150 mg·mL^−1^), the crystal burst from the mid-section of the ice crystal normal to the *c*-axis (Fig. 2d), as is seen with hyperactive AFPs. The two burst patterns are also shown as the Supplementary Movie.

### BpAFP adsorbs to multiple ice planes at high concentrations

**Figure 3:**
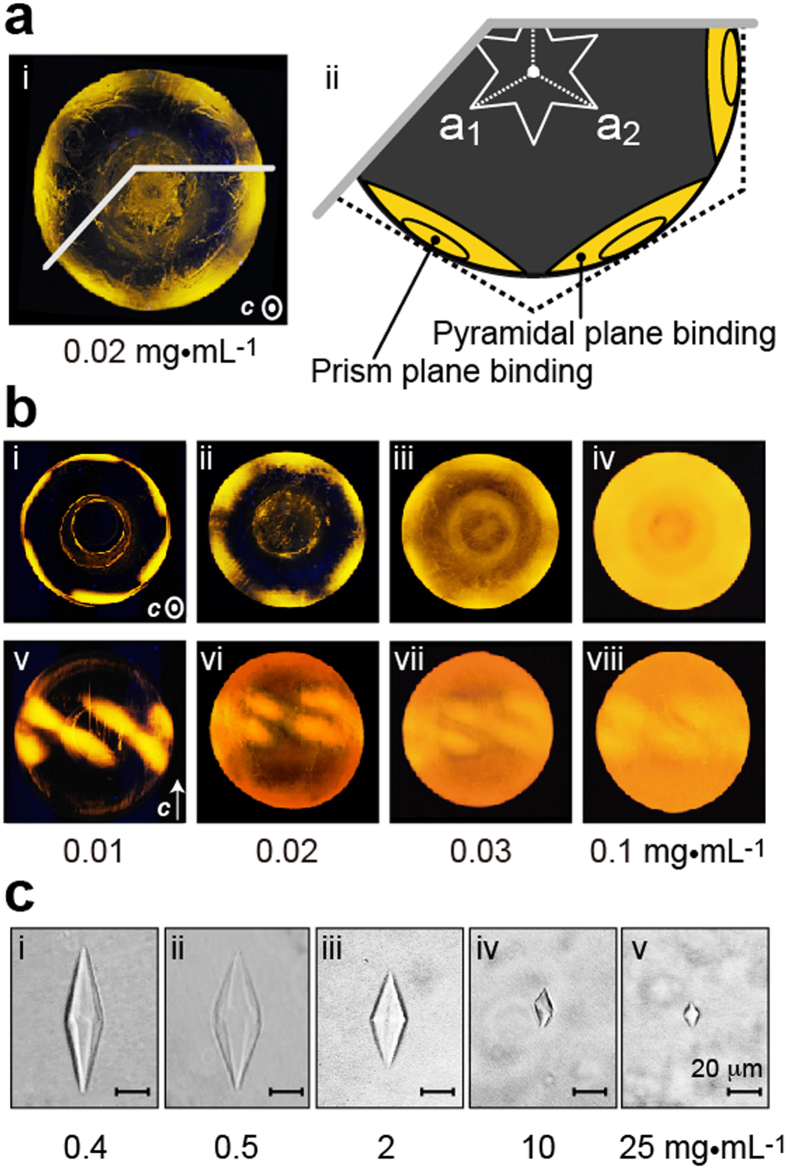
Ice plane affinity of bpAFP: (**a**) Fluorescence-based ice plane affinity (FIPA) analysis of rhodamine^®^-tagged-bpAFP at a concentration of 0.02 mg·mL^−1^ (i) and its interpreted illustration (ii). The direction of the *c*-axis out of the plane of the figure is indicated by the white dot in the circle. (**b**) Change of the FIPA pattern with increasing bpAFP concentration. Upper (i–iv) and lower panels (v–viii) show top and side views of an ice hemisphere, respectively, as guided by *c*-axis direction. (**c**) Change of the ice crystal morphology with increasing the concentration of rbpAFP, in which the scale bars represent 20 μm.

The reason hyperactive AFPs direct ice growth along *a*-axes at the end of TH, is that they adsorb to the basal planes of the ice crystal and block growth along the *c*-axis. FIPA analysis^14^ was performed to identify the bpAFP-bound ice planes on a single ice crystal hemisphere (d = 2.5 cm). At a low bpAFP concentration of 0.01 mg·mL^−1^ we observed illumination from the fluorescently tagged protein in six patches near the periphery of the ice hemisphere when this was oriented with its *c*-axis parallel to the central cold finger (Fig. 3a,i and b,i). When the hemisphere was oriented at right angles to this direction, with the *c*-axis of the ice mounted perpendicular to the cold finger, six of the predicted twelve ellipses were illuminated on the equator of this ice hemisphere (Fig. 3b,v) indicating the bpAFP-binding planes. Note: the middle two ellipse pairs overlap at the equator, as would the other four pairs if the single ice crystal was spherical. Winter flounder AFP (HPLC-6) showed a similar, but not identical pattern of ellipses oriented in the same direction^12^. Its binding surface is the (20–21) pyramidal plane with clear breaks between the bound surfaces on opposite sides of the equatorial line. However, the pyramidal planes bound by bpAFP are less well separated as though they bind to a pyramidal plane that is closer to the primary prism surface. At this low concentration there was no sign of bpAFP or rbpAFP (Supplementary Figure S2) binding to the (0001) basal planes.

With increasing bpAFP concentration the ice hemisphere became illuminated over its entire surface, partially obscuring the plane-specific signals seen at one tenth the highest concentration tested (Fig. 3b,i-iv and b,v-viii). A fish type III AFP does not show any such illumination (Supplementary Figure S3). These observations suggest that bpAFP is capable of binding to multiple ice planes of an ice crystal in a concentration-dependent manner. The bpAFP-binding to the basal plane was clearly demonstrated by illumination at the center of the ice hemisphere mounted with its *c*-axis normal to the cold finger (Fig. 3b,Iv and Supplementary Figure S2b,iv). Widespread binding over the ice hemisphere is typically observed with hyperactive AFPs at a concentration of 0.1–1.0 mg·mL^−1^ 14.

One more indication of hyperactivity came from the ice crystal morphology with increasing bpAFP concentration from 0.4 to 25 mg·mL^−1^ (Fig. 3c). The length of the starting ice crystal along the *c*-axis gradually decreased from 45 to 5 μm over this 60-fold increase in bpAFP concentration. Concomitantly, the hexagonal trapezohedron became less well faceted and rounder in shape, and finally changed into a tiny lemon-like shaped ice crystal. This change in ice crystal morphology also supports the binding of bpAFP to multiple ice planes. Hyperactive AFPs from insects and fish direct similar lemon-shaped ice crystal formation^3^.

### BpAFP is pH Stable and Fully Renaturable

**Figure 4:**
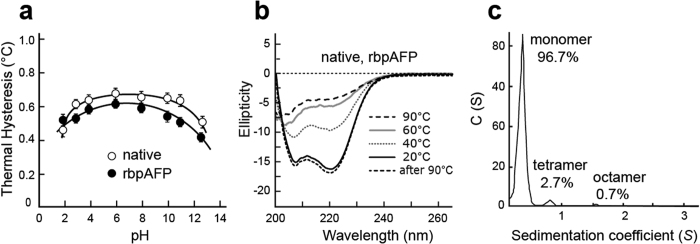
Physical properties of bpAFP: (**a**) TH activity of 10 mg·mL^−1^ of bpAFP and rbpAFP at pH values from 2 to 13. (**b**) The CD spectra of 0.5 mg·mL^−1^ of bpAFP obtained at 20, 40, 60 and 90 °C. The dashed line in the bottom shows the spectrum at 20 °C after prior heating to 90 °C. (**c**) Sedimentation profile of bpAFP from the analytical ultracentrifuge analysis.

The TH activity of rbpAFP and bpAFP (10 mg·mL^−1^) was assayed over the pH range 2 to 13 (Fig. 4a). As seen in Fig. 2(a) the native bpAFP (isoform mixture) was about 10% more active than rbpAFP. Their TH values only slightly decreased towards the acidic end (pH = 2–3) and only slightly more so towards the basic conditions (pH = 11–13), illustrating the stable nature of bpAFP against pH change. A similar stability nature was reported for winter flounder AFP^29^.

An aqueous solution of bpAFP (0.5 mg·mL^−1^) was examined by CD spectroscopy at temperatures between 20 and 90 °C (Fig. 4b). There were two minima at 208 and 222 nm in the CD profile at 20 °C, which clearly indicates the dominance of α-helix structure. The lower minimum at 222 nm is consistent with the slightly over-wound alanine-rich helices seen in type I AFPs, where there are 3.7 residues per turn rather than 3.6. The profile became flatter with heating to 60 °C and above. At this dilution, no aggregation of rbpAFP occurred after heating to 90 °C and cooling, and the tracing of ellipticity vs wavelength returned to the starting profile seen at 20 °C. The CD profiles for native bpAFP were identical to those rbpAFP. Winter flounder AFP^29^ and another type I AFP from cunner^30^,^31^ exhibited a similar thermo-recoverable nature, for which *T*_m_ values of approximately 30 °C were estimated from the CD measurements.

### Analytical Ultracentrifugation Shows BpAFP Tetramer Formation

To see if bpAFP had any tendency to form oligomers in solution, we examined a sample by analytical ultracentrifugation. At a concentration 5 mg·mL^−1^, about 2.7% of the loaded bpAFP sedimented as a tetramer. There was no sign of a dimer or trimer, but a trace amounts of an octamer (Fig. 4c).

## Discussion

We have discovered a 40-residue type I AFP from barfin plaice, which uniquely shows two forms of ice binding at different concentrations. Both bpAFP and rbpAFP bound to pyramidal planes of an ice crystal below 0.01 mg·mL^−1^, while exhibiting additional binding onto the whole ice crystal surface, including the basal planes, above 0.1 mg·mL^−1^. Consistent with this concentration-dependent difference in ice-binding behavior, the morphology of an ice crystal in the presence of the AFP was gradually changed from a hexagonal trapezohedron with distinct facets to a smaller less distinct lemon-shape as the bpAFP concentration increased. The extreme water solubility (~650 mg·mL^−1^) of this alanine-rich protein and its transition from moderately active to hyperactive at high concentrations are exceptional properties not seen in other AFPs.

The CD profile of bpAFP, with two negative minima at 208 and 222 nm, and a high sequence identity with the other type I AFPs has enabled us to predict the monomeric α-helical structure of bpAFP with considerable confidence. The N- and C-terminal capping sequences are the same as in winter flounder type I AFP^17^,^18^. So too are the four Thr (T^2^, T^13^, T^24^, and T^35^) with their 11-residue periodicity that will project the side-chains in a line along the alanine-rich helix. The alanine content of bpAFP is exceptionally high (75%), compared with that of other type I AFPs, such as wfAFP (62%), Alaskan plaice AFP (65%), and yellow-tail flounder AFP (69%)^32^. Aside from the cap structures and the four Thr, the only non-Ala residues are K^26^ and E^30^, which will presumably form a salt-bridge similar to that between K^18^ and E^22^ in wfAFP. We speculate that bpAFP has water-holding properties similar to the 33-kDa type I AFP isoform (Maxi) that organizes ~400 water molecules in the interior of its four-helix bundle structure^23^. Both Maxi and bpAFP are largely composed of the 11-residue repetitive unit TX_10_ (where X is mostly alanine), and the repetitive unit of Maxi forms three helical turns with an average of 3.7 residues per turn, as opposed to 3.6 residues per turn in the classic α-helix^22^,^23^. As shown by the Maxi crystal structure this deviation in the peptide bond facilitates hydrogen bonding between the backbone carbonyl groups and the solvent water molecules. Moreover, the small size of the Ala side chain also helps water to access the peptide backbone. These two factors contribute to the extraordinarily high solubility of bpAFP.

The ice-binding site (IBS) of winter flounder type I AFP has been defined as its alanine-rich face including A^6^, A^17^, and A^28^ along with a flanking row of Thr on one side and Ala on the other. The same ice-binding surface is present in bpAFP, and yet at low concentrations these two AFPs bind to slightly different pyramidal planes^12^. These subtleties are likely influenced by differences in neighboring residues around the helix. We note that the similar Ala-rich AFPs from sculpins bind to the (2-1-10) secondary prism plane of ice^12^.

The hydrophobicity of the ice-binding face of type I and other AFPs^1^ was a key observation in formulating the clathrate water mechanism for adsorption of these AFPs to ice, first proposed by molecular modeling^22^,^32^,^33^. The hydrophobic groups can project water into a cage structure that matches waters in ice and the quasi-liquid layer immediately above the ice surface. Robust evidence for this ice-like water structure came from X-ray crystallography^21^ with the added discovery that these clathrates are stabilized (anchored) to nearby hydrogen bonding groups on the peptide backbone (as in type I AFP) and to hydrophilic side chains. Given that anchored clathrate waters on the ice-binding site can merge with the quasi-liquid layer they can also fuse with waters on another IBS. This compatibility is perhaps one reason why so many AFPs have crystallized with their IBSs face-to-face that has obscured the full extent of their water coverage^23^. A striking example of water-mediated fusion is the structure of Maxi, an extreme variant of type I wfAFP that forms a four-helix bundle structure held together by an internal network of clathrate waters^23^. Tellingly, the ice-binding residues (Thr, Ala, Ala) project inwards to organize the water network that binds the helices together. Despite this, Maxi has hyperactive ice-binding activity, which is explained by the water organization extending outwards from the interior to bind the AFP to many different planes of ice, including the basal planes.

We suggest this same process is at work here with bpAFP. A small percentage of the monomer associates in a concentration-dependent manner, presumably through its ice-binding site, to form a tetramer which, like Maxi, has a more extensive array of clathrate waters on its outer surface to match multiple planes of ice. This hypothesis explains why bpAFP has the properties of moderately active type I AFP at low concentrations, and hyperactivity at higher concentrations. Indeed, it is quite possible that Maxi evolved along these lines in order to help stabilize these tentative beneficial associations of four helices. By extending the length of the helix through repeated gene duplication, and with folding into a hairpin that can dimerize in an antiparallel way through clathrate waters, a more stable four-helix bundle structure would be achieved. We note that the antiparallel alignment of four bpAFP helices has the potential for electrostatic interactions between the N- and C-terminal cap structures, with two positive and two negative charges at either end.

## Materials and Methods

### Purification of native bpAFP from muscle homogenate

Barfin plaice (Fig. 1a) were caught in the Okhotsk coastal area of Hokkaido in 2014 February. Fillets were cut from the fish and ground to make a muscle paste. We then prepared a suspension of 2 kg of this paste in 2 L of water, which was ultrafiltered (Pellicon XL Biomax 30 kDa, Merck Millipore, Germany). The ultrafiltrate was lyophilized to obtain 6 g of proteins with a molecular weight of less than 30 kDa. This sample was dissolved in 20 mL of 20 mM Tris-HCl buffer (pH 8.5) containing 0.2 M NaCl. Following centrifugation at 9,000×g for 20 min, the supernatant was loaded onto a Sephadex G-25 size-exclusion column (XK 50/30, 500 mL, GE healthcare, USA). The void-volume fraction that exhibited the antifreeze activity was loaded onto a DEAE Sepharose anion-exchange column (XK 50/20, 80 mL, GE healthcare, USA) equilibrated with 20 mM Tris-HCl buffer (pH 8.5). We then collected 120 mL of initial flow-through fraction using the same buffer (Fig. 1b), which contained the barfin plaice AFP with 90–95% purity (Fig. 1c). This eluate was dialyzed against distilled water to remove the buffer, and was lyophilized to obtain approximately 1 g of the final product. Repetition of these procedures produced gram-quantities of this AFP.

### Expression of bpAFP and fluorescence labeling

The DNA encoding Trx-tagged bpAFP (Fig. 1f) was cloned into expression vector pET32a in *Escherichia coli* BL21 (DE3) Rosetta2 (Novagen). These cells in Luria-Bertani medium were harvested by centrifugation, washed by resuspension with 50 mM Tris-HCl buffer (pH 8.0) for disruption by sonication. The lysate supernatant was applied to Ni-NTA column to obtain the Trx-tagged bpAFP. This fusion protein (50 mg protein in 30 mL of PBS) was digested with thrombin at 22 °C for 16 h, and the products were applied to a Ni-NTA column to retain cleaved Trx and uncleaved fusion protein. The flow-through material was then applied to TSKgel ODS-80Ts (TOSOH) for reversed-phase HPLC, which produced an rbpAFP sample of 98% purity (Supplementary Figure S1b). Average yield of rbpAFP was approximately 25 mg per L of culture medium.

Native and recombinant samples of bpAFP were fluorescently tagged for FIPA experiments. Native or recombinant bpAFP (4 mg) in 1 mL of 0.1 M ammonium bicarbonate (pH 7.9) was allowed to react with 50 μL of 5.0 mg·mL^−1^ of Rhodamine (Life Technologies) by mixing in a tube rotator at 25 °C for 4 h. Following removal of unreacted dye by ultrafiltration, 0.01, 0.02, 0.03, and 0.1 mg·mL^−1^ dilutions of the fluorescent peptide were prepared for FIPA analysis.

### Measurements of TH, FIPA, and CD spectra

TH activity was measured with a LEICA DMLB100 photomicroscope (Leica Microsystems, Wetzlar, Germany) equipped with a Linkam THMS 600 temperature controller (Linkam Scientific Instruments Ltd, Tadworth, Surrey, UK) according to procedures described in ref. 34. Briefly, aqueous sample (0.8 μL) at the concentrations of 1, 2, 5, 10, 15, 20, 30, 50, 60, 75, 100, 150, and 200 mg·mL^−1^ was frozen in the photomicroscope apparatus and melted back to prepare a single seed ice crystal. Its bursting-growth and melting temperatures were determined as *T*_f_ and *T*_m_, respectively. This measurement was repeated at least 3 times to average the values for TH activity (TH = |*T*_f_ − *T*_m_|) shown in Figs 2 and 4.

For the FIPA experiments, a single ice crystal hemisphere of 2.5 cm in diameter was prepared and its *c*-axis identified^14^. This ice hemisphere was attached to a cooling probe in a desired orientation, and immersed in *ca.* 30 mL solution of the fluorescent bpAFP. Following slow growth for 120 min, the ice hemisphere was detached from the probe, rinsed to remove any contaminants non-specifically attached to the hemisphere, illuminated under UV light and photographed at −1 °C.

CD measurement were performed for 0.01, 0.02, 0.03, 0.06, 0.08, 0.1, 0.2, 0.3, 0.4, and 0.5 mg·mL^−1^ solutions of native and recombinant bpAFP dissolved into 10 mM sodium phosphate buffer (pH 7.4) using a JASCO J-725 CD spectrophotometer (Jasco Analytical Instruments, MD, USA). The samples were set to a temperature between 20 and 90 °C at intervals of 5 °C, and the results were expressed as mean residue ellipticity (deg·cm^2^·dmol^−1^) at each wavelength (Fig. 4b and Supplementary Figure S4).

### Analytical ultracentrifugation (AUC)

A Beckman Optima XL-I Analytical ultracentrifuge (Beckman Coulter) was used for sedimentation velocity measurements at 40,000 rpm at 4 °C with 2.5 mg of bpAFP in 0.5 mL AFP of 10 mM sodium phosphate (pH 7.9). Double sector charcoal-Epon cells equipped with quartz windows were used and concentration distributions were determined by absorbance optics. Sedimentation coefficient distributions were determined using standard methodology

## Additional Information

**How to cite this article**: Mahatabuddin, S. *et al*. Concentration-dependent oligomerization of an alpha-helical antifreeze polypeptide makes it hyperactive. *Sci. Rep.* **7**, 42501; doi: 10.1038/srep42501 (2017).

**Publisher's note:** Springer Nature remains neutral with regard to jurisdictional claims in published maps and institutional affiliations.

## Supplementary Material

Supplementary Movie 1

Supplementary Materials
