# Supplementary material for: Concentration-dependent oligomerization of an alpha-helical antifreeze polypeptide makes it hyperactive
Source: Sci Rep. 2017 Feb 13;7:42501. doi: 10.1038/srep42501 (PMC5304152; doi:10.1038/srep42501)
Supplement: Supplementary Materials [file srep42501-s2.pdf]

Supplementary Information of

Concentration-dependent oligomerization of an alpha-helical antifreeze polypeptide makes it hyperactive

Sheikh Mahatabuddin[a], Yuichi Hanada[a], Yoshiyuki Nishimiya[b], Ai Miura[b], Hidemasa Kondo[a,b], Peter L. Davies[c] & Sakae Tsuda[a,b] *

[a]Graduate School of Life Science, Hokkaido University, Sapporo 060-0810, Japan.
[b]Bioproduction Research Institute, National Institute of Advanced Industrial Science and Technology (AIST), Sapporo 062-8517, Japan.
[c]Protein Function Discovery Group and Department of Biomedical and Molecular Sciences, Queen's University, Kingston, ON, Canada K7L 3N6.

Supplementary Figure S1

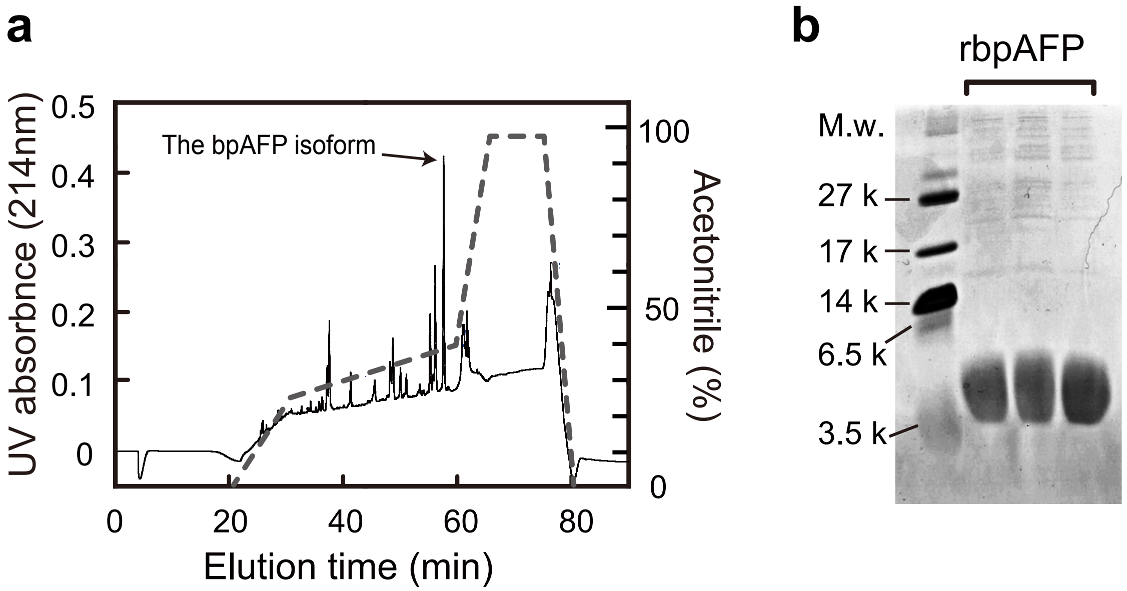

Supplementary Figure S1. Preparation of bpAFP sample. (**a**) Reversed-phase HPLC chromatogram (TSKgel ODS-80Ts column, TOSOH, Tokyo, Japan) of native isoform mixture of barfin plaice AFP. The bpAFP isoform was eluted by a linear gradient of 0-100% acetonitrile in 0.1% trifluoroacetic acid (dashed line). (**b**) Electrophoretogram of the recombinant bpAFP (rbpAFP) separated by 15 % tricine SDS-PAGE with molecular weight markers indicated on the left.

Supplementary Figure S2

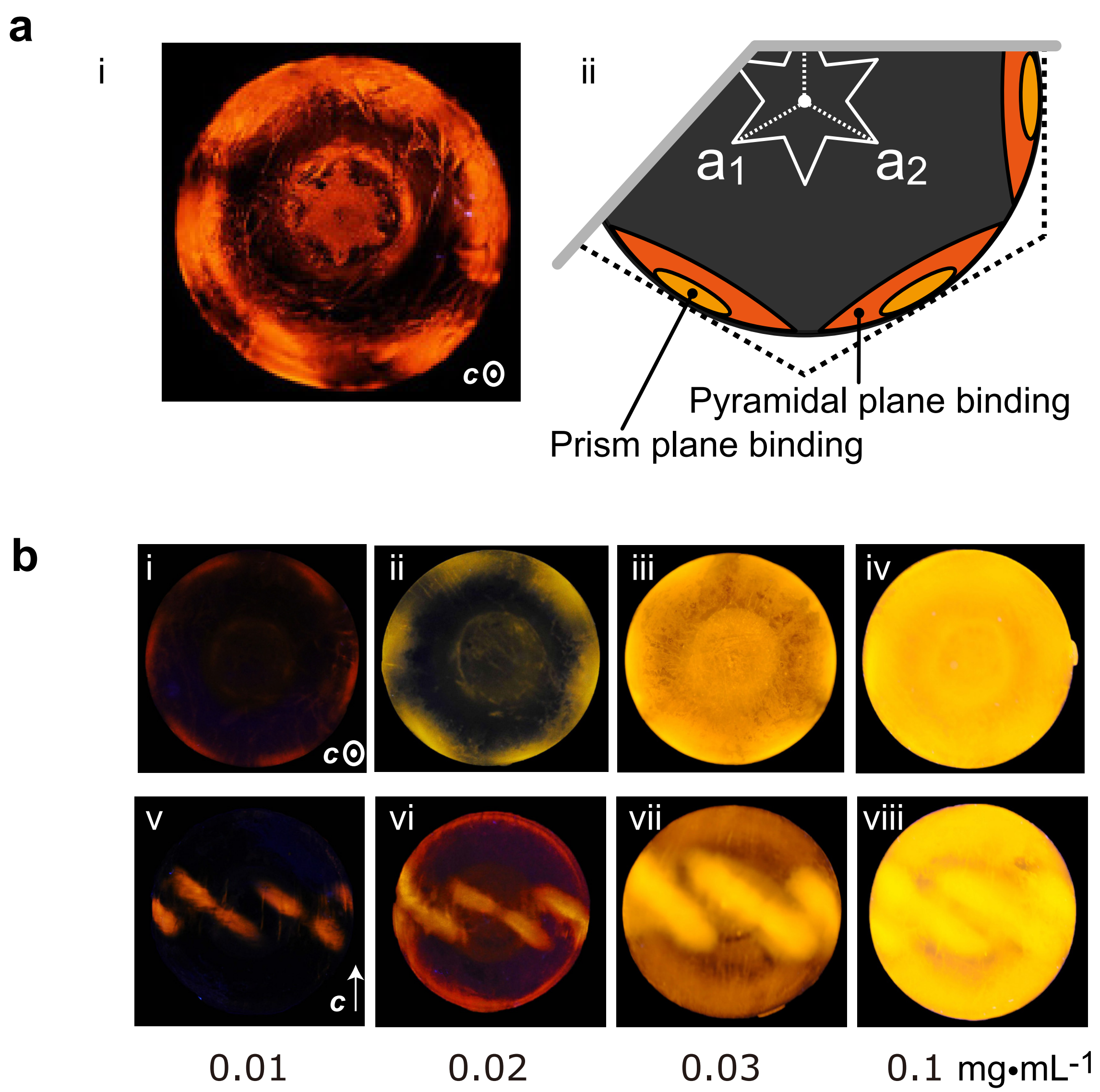

Supplementary Figure S2. Ice-binding affinity of rbpAFP. (**a**) The fluorescence- based ice plane affinity (FIPA) of rhodamine®-tagged rbpAFP at a concentration of 0.02 mg·mL-1 (i), and its interpreted illustration (ii). (**b**) Change of the FIPA pattern with increasing the rbpAFP concentration. Upper (i-iv) and lower panels (v-viii) show top and side views of an ice hemisphere, respectively. The direction of the *c*-axis is shown by the white circle (upper) and arrow (lower).

Supplementary Figure S3

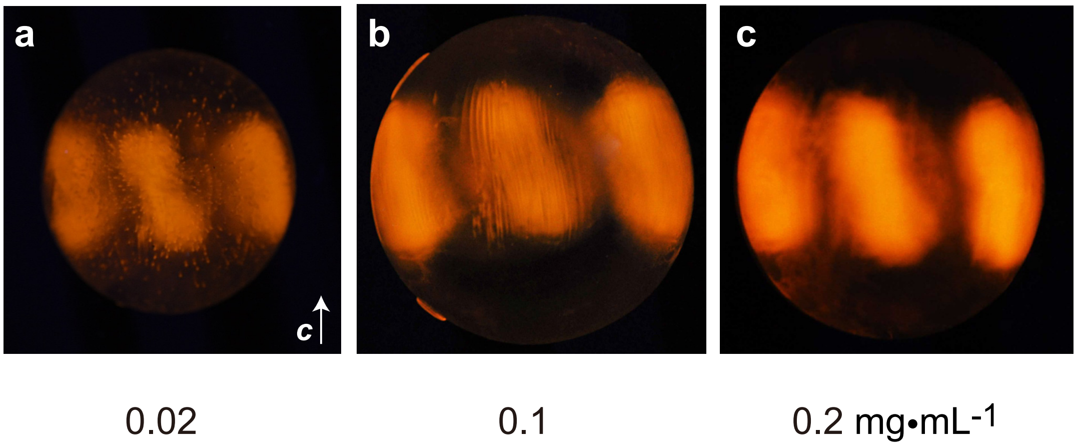

Supplementary Figure S3. Fluorescence-based ice plane affinity (FIPA) analysis of recombinant nfeAFP6 isoform of type III AFP from Notched-fin eelpout, *Zoarces elongatus Kner*. Unlike bpAFP, nfeAFP6 does not bind to the whole ice hemisphere surface even at a concentration of 0.2 mg/mL. The direction of the *c*-axis is shown by the white arrow.

Supplementary Figure S4

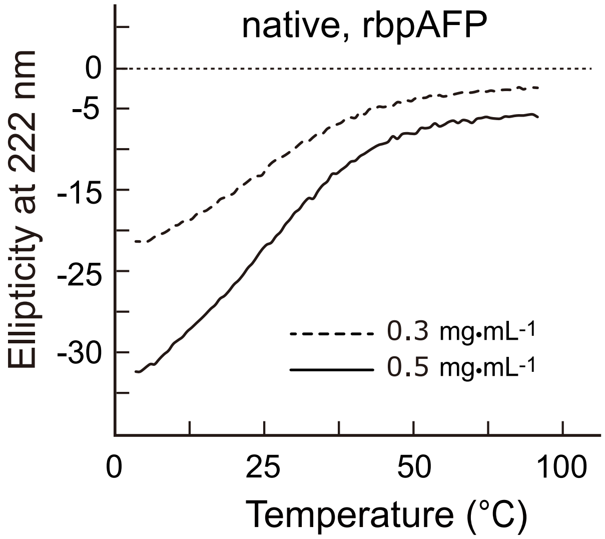

Supplementary Figure S4. Change of the mean residue ellipticity at 222 nm of native- and recombinant bpAFP as a function of temperature. The same profile was obtained for these peptides at the concentrations of 0.01, 0.02, 0.03, 0.06, 0.08, 0.1, 0.2, 0.3, 0.4, and 0.5 mg•mL-1, suggesting that bpAFP undergoes no critical transition between monomer and oligomer.

Supplementary Movie 1

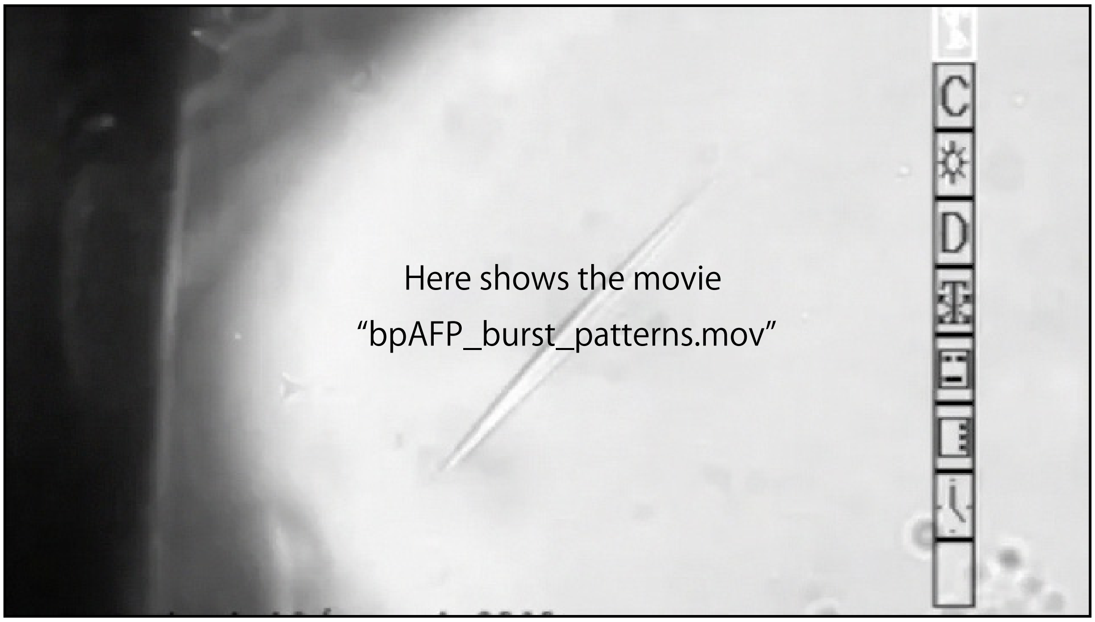

Supplementary Movie 1. Movie files showing two different patterns of bursting ice crystal growth from an ice bipyramid in the presence of recombinant bpAFP (rbpAFP). The first 6-second movie shows a crystal bursting along with the c-axis of the ice bipyramid in 5 m/mL rbpAFP, and in the last 6-second movie the burst pattern in 150 mg/mL rbpAFP is normal to the *c*-axis. Figure 2 in the main text shows four snapshots from each pattern.
